# Supplementary material for: Overcoming Barriers: Strategies for Implementing Pharmacist-Led Pharmacogenetic Services in Swiss Clinical Practice
Source: Genes (Basel). 2024 Jul 1;15(7):862. doi: 10.3390/genes15070862 (PMC11276441; doi:10.3390/genes15070862)
Supplement: Supplementary file 1 [file genes-15-00862-s001.zip › Supplement S1.pdf]

Supplement S1: Semi-Structured Interview Guide

Supplement 1 contains the interview guides for pharmacists and physicians in English. The interviews were conducted using a German-language interview guide.

Semi-Structured Interview - Pharmacists

Introduction
Hello!
I am delighted that you have chosen to participate in this interview, which is part of my master's thesis. Before we begin the interview, I would like to provide you with some basic information about my master's thesis.

Current Legal Framework
On December 1, 2022, the revised Regulation on Genetic Testing in Humans (GUMV) came into force. According to Article 7, you are now authorized as a pharmacist to initiate and conduct pharmacogenetic testing yourself.

What am I investigating during my master's thesis?
The aim of my master's thesis is to identify and develop strategies that promote the implementation of pharmacogenetics as a service in pharmaceutical practice. To develop these strategies, I have decided to conduct interviews with interested pharmacists.

Why were you selected as an interviewee?
As a pharmacist, your personal professional experience and expertise can provide valuable and helpful information in developing possible strategies. I am eager to hear what you will share with me during the interview.

What is expected of you?
Through the interview, I aim to understand your personal opinion or experience. There are no right or wrong answers. The interview lasts approximately 45 minutes and mainly consists of open-ended questions. The interview is composed of 7 main themes, with each theme consisting of approximately 6 questions. In total, the interview comprises 41 questions. I kindly ask you not to mention any names (yours, employer, team members) during the interview to ensure the anonymity of those involved.

Recording of the Interview
Please note that the conversation during the interview will be recorded as an audio file. The recording is intended to ensure that your responses can be accurately transcribed for subsequent analysis. Your responses will be documented in a coded manner in writing. If the collected data is published in the future, it will not be traceable back to you as an individual. The audio recordings will be deleted no later than the end of the master's thesis in June 2023.

Confidentiality
A confidentiality agreement was signed at the beginning of the master's thesis. This confidentiality agreement remains in effect even after the completion of the master's thesis.

Declining Participation
You may withdraw from participating in the interview at any time. If any question makes you uncomfortable, you are welcome to refuse to answer without providing a reason.

If you agree with the above information and have no further questions or uncertainties, we will proceed with the interview.

I look forward to your responses and sincerely thank you for your participation!

1 Questions about the Person
At the beginning of the interview, I would like to gather basic information about your professional practice and the institution where you currently work. Answers can be very brief; the information will be considered for analyzing the responses.

1.1 What type of pharmacy are you currently working in?
- o Chain
- o Grouping
- o Independent
- o Hospital pharmacy
- o Other:__________________________ (please specify)
- o Not currently working in a pharmacy

1.2 In what category would you classify the pharmacy where you currently work?
- o Rural pharmacy
- o Neighborhood pharmacy (i.e., located more on the outskirts of the city)
- o City pharmacy (i.e., located more in the city center)

1.3 Is the pharmacy where you currently work located in a self-dispensing canton?
- o Yes
- o No
- o Mixed form
- o Not currently working in a pharmacy

1.4 How long have you been practicing your profession? (Please specify in months or years)

1.5 What is your current position?
- o Employee
- o Deputy management
- o Management

1.6 How long have you been practicing your profession in your current position at the current institution? (Please specify in months or years)

2 General Process

You have initiated a collaboration with the company "YAI" and/or you have attended the course "Pharmacogenetics in Pharmacy".

2.1 What considerations have you already made regarding "Pharmacogenetics as a service in the pharmacy"?

Now, I'm interested in the current situation at your institution.

2.2 Do you want to implement pharmacogenetics as a service in your institution?

2.2.1 If yes, why? What is your motivation? / If no, why not?

2.3 Have you already begun implementing pharmacogenetic services in your institution?

2.3.1 In your opinion, what are the greatest challenges in implementing pharmacogenetics as a service?

2.4 Do you think the infrastructure in your pharmacy is currently equipped enough to carry out pharmacogenetics in daily practice?

2.5 How should the team be involved in the implementation process?

3 Time Investment

In this section, I'm interested in your personal opinion regarding the time investment required for conducting pharmacogenetic services in your institution.

3.1 How much time would you estimate per patient to initiate, conduct, and evaluate the results of a pharmacogenetic test?

3.2 How do you assess your time capacities/the time capacities of the pharmacist for offering and conducting pharmacogenetic tests?

3.2.1 What is the maximum time you can allocate per patient to conduct a pharmacogenetic test in your institution?

3.3 In your opinion, how should the process be organized in your institution/pharmacy to make the implementation of pharmacogenetic testing feasible?

4 Patient

During the implementation of pharmacogenetics in pharmaceutical practice, the needs and expectations of patients will influence the success of the implementation process. Therefore, we will now focus more closely on patients.

4.1 Do you think your patients would be interested in pharmacogenetic services at your institution?

4.2 In your opinion, what are the most common reasons for patients to agree to a pharmacogenetic test?

4.3 In your opinion, what are the most common reasons for patients to refuse a pharmacogenetic test?

4.4 How do you proceed/would you proceed to identify patients who might benefit from a pharmacogenetic test?

According to the Federal Act on Human Genetic Testing (GUMG), certain principles must be adhered to when conducting pharmacogenetic tests in your institution. According to Article 5 and Article 6 of the GUMG, genetic testing may only be carried out if the patient consents freely and willingly after comprehensive and understandable information. Among other things, the patient should be informed about the benefits and limitations of pharmacogenetic testing, the handling of the sample and genetic data, as well as their own rights.

4.5 In your opinion, what should be provided to the patient to ensure they are sufficiently informed (and subsequently consent)?

4.6 What hurdles might arise when informing the patient?

5 Costs

Currently, with the exception of a few specific medications, pharmacogenetic tests are not covered by mandatory health insurance unless ordered by a pharmacologist.

5.1 How does this fact affect the implementation of pharmacogenetic services in your institution?

5.2 What do you think a patient would be willing to pay?

5.3 What do you aim to earn per analysis? Could you please provide an estimate?

6 Interprofessional Relationships

Experiences from the Pharmaceutical Care Research Group Basel have shown that interprofessional collaboration is a fundamental requirement for successful implementation of pharmacogenetics.

6.1 What are your experiences so far in collaborating with physicians in conducting a pharmacogenetic test?

6.2 How do you envision collaborating with physicians in conducting a pharmacogenetic test?

6.3 What could complicate collaboration with physicians in conducting a pharmacogenetic test? What obstacles do you see?

6.4 What could facilitate collaboration with physicians in conducting a pharmacogenetic test?

According to Article 7 of the Regulation on Genetic Testing in Humans (GUMV), pharmacists are bound by certain obligations when initiating a pharmacogenetic test. If the initiation of a pharmacogenetic test is intended to obtain insights that might warrant a change in medication therapy, the prescribing physician must first be informed of this intention.

Imagine you deem it appropriate to conduct a pharmacogenetic test on a patient. You now want to inform the prescribing physician about your intentions.

6.5 In your opinion, how should contact be made with the prescribing physician so that you can initiate a pharmacogenetic test?

With the following questions, we will discuss the exchange of patient-related data. Currently, Switzerland does not have a uniform e-health strategy.

6.6 How do you proceed/would you proceed to inform the prescribing physician of the results of the genetic analysis?

6.7 What do you pay attention to/what would you pay attention to when communicating the results to ensure successful collaboration with the prescribing physician?

Data from the literature show that pharmacogenetic interventions by pharmacists are implemented by the physician only about 60% of the time.

6.8 In your opinion, what general factors might lead the physician not to implement all pharmacogenetic recommendations made by the pharmacist?

If a pharmacogenetic test is conducted in the pharmacy, you will commission a laboratory to analyze the genetic sample.

6.9 What possible obstacles do you see/What are your experiences in selecting and collaborating with the laboratory?

6.10 What factors, in your opinion, simplify the selection and collaboration with the laboratory?

7 Knowledge

Initiating a pharmacogenetic test in the pharmacy requires specific expertise to interpret the results of the genetic analysis and to develop concrete therapy recommendations based on them.

7.1 How do you assess your current level of knowledge of pharmacogenetics?

7.2 What sources of information would you like to have to enrich your knowledge of pharmacogenetics?

7.3 To whom or to which source of information would you prefer to turn in case of complicated questions during the execution and analysis of a pharmacogenetic test?

When implementing pharmacogenetics as a service in your institution, you will likely involve your team.

7.4 How do you assess the current knowledge of your team on the topic of pharmacogenetics?

7.5 How have you proceeded/will you proceed to train your team on the topic of pharmacogenetics?

Closing Remarks
That was the last question I wanted to discuss with you. Thank you very much for your participation!

Semi-Structured Interview - Physicians

Introduction
Hello!
I am delighted that you have chosen to participate in this interview, which is part of my master's thesis. Before we begin the interview, I would like to provide you with some basic information about my master's thesis.

Current Legal Basis
On December 1, 2022, the revised Regulation on Genetic Testing in Humans (GUMV) came into force. According to Article 7, pharmacists are now authorized to conduct pharmacogenetic tests in their pharmacies.

What am I investigating during my master's thesis?
The aim of my master's thesis is to identify and develop strategies that promote the implementation of pharmacogenetics as a service in pharmaceutical practice.
From experiences in previous research projects, we know that interprofessional relationships between pharmacists and physicians make a significant contribution to a successful implementation process. This has led me to conduct interviews with interested physicians. I am eager to hear what you will share with me during the interview.

What is expected of you?
Through the interview, I aim to understand your personal opinion or experience. There are no right or wrong answers. The interview lasts approximately 30 minutes and mainly consists of open-ended questions. The interview consists of 6 main themes, with each theme consisting of approximately 4-5 questions. In total, the interview comprises 28 questions. I kindly ask you not to mention any names (yours, employer, team members) during the interview to ensure the anonymity of those involved.

Recording of the Interview
Please note that the conversation during the interview will be recorded as an audio file. The recording is intended to ensure that your responses can be accurately transcribed for subsequent analysis. Your responses will be documented in a coded manner in writing. If the collected data is published in the future, it will not be traceable back to you as an individual. The audio recordings will be deleted no later than the end of the master's thesis in June 2023.

Confidentiality
A confidentiality agreement was signed at the beginning of the master's thesis. This confidentiality agreement remains in effect even after the completion of the master's thesis.

Declining Participation
You may withdraw from participating in the interview at any time. If any question makes you uncomfortable, you are welcome to refuse to answer without providing a reason.

If you agree with the above information and have no further questions or uncertainties, we will proceed with the interview.

I look forward to your responses and sincerely thank you for your participation!

1 Questions about the Person
At the beginning of the interview, I would like to gather basic information about your professional practice and the institution where you currently work. Answers can be very brief; the information will be considered for analyzing the responses.

1.1 What type of institution are you currently working in?

- General practitioner's office
- Outpatient psychiatry
- Inpatient psychiatry
- Hospital
- Other: ____ (please specify)

1.2 Is the institution where you currently work located in a self-dispensing canton?
- Yes
- No
- Mixed form

1.3 How long have you been practicing your profession? (Please specify in months or years)

1.4 What is your current position?

1.5 How long have you been practicing your profession in your current position at the current institution? (Please specify in months or years)

2 General Process

With the current legal framework, pharmacists are now empowered to conduct pharmacogenetic tests in the pharmacy.

2.1 What considerations have you already made regarding the topic of "Pharmacogenetics as a service in pharmacies"?

I am now interested in your previous experiences on this topic.

2.2 Have you already used a pharmacogenetic service provided by a pharmacist?

2.2.1 If yes, why? What were your motivating factors? / If no, why not yet?

2.3 In your opinion, what are the greatest challenges in implementing pharmacogenetics as a service in pharmacies?

4 Patient

During the implementation of pharmacogenetics in pharmaceutical practice, the needs and expectations of patients will influence the success of the implementation process. Therefore, we will now focus more closely on patients.

4.1 Do you think your patients would be interested in pharmacogenetic services at your institution?

4.2 In your opinion, what are the most common reasons for patients to agree to a pharmacogenetic test?

4.3 In your opinion, what are the most common reasons for patients to refuse a pharmacogenetic test?

4.4 How do you proceed/would you proceed to identify patients who might benefit from a pharmacogenetic test?

According to the Federal Act on Human Genetic Testing (GUMG), certain principles must be adhered to when conducting pharmacogenetic tests in your institution. According to Article 5 and Article 6 of the GUMG, genetic testing may only be carried out if the patient consents freely and willingly after comprehensive and understandable information. Among other things, the patient should be informed about the benefits and limitations of pharmacogenetic testing, the handling of the sample and genetic data, as well as their own rights.

4.5 In your opinion, what should be provided to the patient to ensure they are sufficiently informed (and subsequently consent)?

4.6 What hurdles might arise when informing the patient?

5 Costs

Currently, with the exception of a few specific medications, pharmacogenetic tests are not covered by mandatory health insurance unless ordered by a pharmacologist.

5.1 In your opinion, how will this fact influence the implementation of pharmacogenetics into pharmaceutical practice?

5.2 What do you think a patient would be willing to pay?

5.3 What do you aim to earn per analysis? Could you please provide an estimate?

6 Interprofessional Relationships

With the following questions, I would like to address the topic of interprofessional relationships between physicians and pharmacists. Experiences from the Pharmaceutical Care Research Group Basel have shown that interprofessional collaboration is a fundamental requirement for successful implementation of pharmacogenetics.

6.1 What are your experiences so far in collaborating with pharmacists in conducting a pharmacogenetic test?

6.2 What could complicate collaboration with pharmacists in conducting a pharmacogenetic test? What obstacles do you see?

6.3 What could facilitate collaboration with pharmacists in conducting a pharmacogenetic test?

According to Article 7 of the Regulation on Genetic Testing in Humans (GUMV), pharmacists are bound by certain obligations when initiating a pharmacogenetic test. If the initiation of a pharmacogenetic test is intended to obtain insights that might warrant a change in medication therapy, you as the prescribing physician must first be informed of this intention.

Imagine the above situation applies now. The pharmacist decides to contact you, as the prescribing physician.

6.4 How should the pharmacist contact you to inform you about the intention to perform a pharmacogenetic test on your patient?

With the following questions, we will discuss the exchange of patient-related data. Currently, Switzerland does not have a uniform e-health strategy.

6.5 How should the pharmacist communicate the results of the genetic analysis to you so that you can consider making a therapy change if necessary?

Data from the literature show that pharmacogenetic interventions by pharmacists are implemented by the physician only about 60% of the time.

6.6 In your opinion, what general factors might lead the physician not to implement all pharmacogenetic recommendations made by the pharmacist?

6.7 What do you specifically expect from the pharmacist for you to consider a therapy change based on the results of the genetic test?

7 Knowledge

You are the prescribing physician. Interpreting the results of the genetic analysis requires specific expertise to develop concrete therapy recommendations based on them.

7.1 How do you assess your current level of knowledge of pharmacogenetics?

7.2 What sources of information would you like to have to enrich your knowledge of pharmacogenetics?

7.3 To whom or to which source of information would you prefer to turn in case of complicated questions during the execution and analysis of a pharmacogenetic test?

Closing Remarks

That was the last question I wanted to discuss with you. Thank you very much for your participation!
