# Supplementary material for: Overcoming Barriers: Strategies for Implementing Pharmacist-Led Pharmacogenetic Services in Swiss Clinical Practice
Source: Genes (Basel). 2024 Jul 1;15(7):862. doi: 10.3390/genes15070862 (PMC11276441; doi:10.3390/genes15070862)
Supplement: Supplementary file 1 [file genes-15-00862-s001.zip › Supplement S2.pdf]

Supplement S2: CFIR-ERIC Implementation Strategy Matching Tool

The results of the CFIR-ERIC Implementation Strategy Matching Tool (cumulative percentage) combined with the ERIC strategies mentioned by pharmacists and physicians in the interviews (absolute number).

ERIC Strategies	Cumulative Percent	Evidence Strength & Quality	Design Quality & Packaging	Patient Needs & Resources	Cosmopolitanism	Peer Pressure	External Policy & Incentives	Structural Characteristics	Networks & Communications	Relative Priority	Access to knowledge & information	Pharmacists	Physicians	Sum (Pharmacists and Physicians)
Conduct local consensus discussions	253%	41%	26%	29%	15%	28%	22%	14%	22%	46%	10%	5	2	7
Identify and prepare champions	222%	41%	15%	5%	15%	38%	22%	27%	17%	18%	24%	0	0	0
Build a coalition	219%	6%	0%	14%	62%	16%	33%	27%	39%	18%	3%	0	0	0
Conduct educational meetings	218%	47%	22%	10%	12%	9%	15%	5%	13%	7%	79%	3	2	5
Capture and share local knowledge	199%	25%	15%	10%	23%	6%	26%	23%	26%	14%	31%	0	0	0
Create a learning collaborative	186%	16%	7%	0%	31%	16%	15%	18%	35%	4%	45%	2	3	5
Involve patients/consumers and family members	185%	13%	19%	71%	4%	28%	11%	9%	9%	18%	3%	0	0	0
Assess for readiness and identify barriers and facilitators	180%	13%	7%	33%	15%	16%	4%	36%	13%	36%	7%	0	0	0
Inform local opinion leaders	175%	38%	19%	0%	15%	25%	22%	14%	22%	14%	7%	0	0	0
Conduct local needs assessment	172%	3%	15%	57%	12%	16%	7%	18%	9%	32%	3%	6	6	12
Promote network weaving	167%	0%	0%	0%	50%	13%	11%	23%	57%	4%	10%	5	3	8
Alter incentive/allowance structures	158%	3%	0%	10%	0%	47%	41%	18%	0%	39%	0%	0	0	0
Develop educational materials	144%	28%	33%	10%	4%	0%	4%	0%	0%	7%	59%	7	7	14
Involve executive boards	140%	6%	4%	5%	23%	28%	41%	14%	9%	11%	0%	1	0	1
Use advisory boards and workgroups	137%	9%	19%	29%	35%	6%	15%	5%	13%	7%	0%	0	0	0
Conduct educational outreach visits	136%	34%	15%	5%	23%	13%	0%	0%	4%	14%	28%	0	0	0
Obtain and use patients/consumers and family feedback	133%	6%	30%	76%	0%	9%	0%	5%	0%	7%	0%	0	0	0
Identify early adopters	130%	22%	11%	0%	4%	28%	7%	23%	17%	7%	10%	0	0	0
Promote adaptability	119%	3%	48%	14%	0%	6%	0%	23%	0%	18%	7%	0	0	0
Develop academic partnerships	118%	25%	4%	5%	50%	0%	11%	5%	9%	0%	10%	5	1	6
Distribute educational materials	118%	31%	19%	5%	0%	0%	0%	0%	4%	4%	55%	5	5	10
Visit other sites	101%	13%	4%	0%	38%	13%	7%	5%	4%	4%	14%	0	0	0
Facilitation	95%	0%	7%	0%	12%	13%	4%	9%	26%	14%	10%	0	0	0
Organize clinician implementation team meetings	93%	3%	4%	0%	0%	3%	0%	14%	52%	4%	14%	0	0	0
Develop a formal implementation blueprint	90%	0%	15%	5%	4%	0%	7%	18%	13%	14%	14%	2	0	2
Tailor strategies	83%	6%	15%	14%	0%	0%	11%	18%	4%	14%	0%	4	0	4
Develop and implement tools for quality monitoring	79%	6%	30%	14%	0%	6%	11%	5%	0%	7%	0%	3	0	3
Provide local technical assistance	77%	3%	4%	5%	4%	3%	7%	18%	9%	0%	24%	0	0	0
Audit and provide feedback	76%	13%	4%	5%	0%	16%	0%	5%	17%	14%	3%	0	0	0
Increase demand	76%	0%	4%	10%	0%	34%	0%	0%	0%	29%	0%	0	0	0
Use an implementation adviser	75%	6%	15%	5%	8%	3%	4%	5%	9%	7%	14%	8	8	16
Recruit, designate and train for leadership	74%	3%	0%	0%	15%	6%	0%	18%	17%	11%	3%	0	0	0
Conduct cyclical small tests of change	72%	3%	11%	10%	0%	6%	4%	23%	9%	4%	3%	0	0	0

Obtain formal commitments	69%	0%	0%	0%	19%	3%	15%	9%	9%	14%	0%	2	0	2
Work with educational institutions	69%	16%	11%	0%	19%	3%	4%	5%	4%	0%	7%	4	4	8
Provide ongoing consultation	68%	9%	7%	5%	0%	6%	0%	9%	0%	14%	17%	0	0	0
Mandate change	65%	0%	0%	0%	0%	9%	15%	5%	4%	32%	0%	0	0	0
Fund and contract for clinical innovation	65%	0%	4%	0%	0%	19%	15%	14%	0%	11%	3%	0	0	0
Model and simulate change	63%	3%	11%	0%	8%	13%	4%	14%	4%	0%	7%	0	0	0
Purposely reexamine the implementation	62%	6%	22%	5%	4%	6%	11%	0%	4%	4%	0%	0	0	0
Use mass media	61%	6%	0%	0%	8%	13%	15%	0%	9%	7%	3%	1	1	2
Conduct ongoing training	60%	6%	4%	0%	0%	0%	4%	0%	4%	4%	38%	6	1	7
Prepare patients/consumers to be active participants	57%	0%	0%	48%	0%	9%	0%	0%	0%	0%	0%	9	9	18
Facilitate relay of clinical data to providers	55%	6%	7%	10%	0%	6%	4%	0%	4%	7%	10%	9	9	18
Create or change credentialing and/or licensure standards	51%	3%	0%	0%	4%	6%	19%	5%	0%	14%	0%	0	0	0
Centralize technical assistance	45%	3%	4%	0%	4%	0%	0%	5%	26%	0%	3%	0	0	0
Intervene with patients/consumers to enhance uptake & ad	45%	3%	7%	24%	0%	3%	4%	0%	0%	4%	0%	0	0	0
Start a dissemination organization	45%	0%	4%	5%	8%	6%	0%	9%	13%	0%	0%	0	0	0
Access new funding	44%	3%	4%	0%	4%	6%	7%	5%	4%	11%	0%	0	0	0
Develop resource sharing agreements	43%	0%	0%	0%	31%	0%	0%	5%	4%	0%	3%	0	0	0
Make training dynamic	39%	6%	11%	0%	0%	0%	0%	5%	0%	7%	10%	0	0	0
Shadow other experts	39%	3%	4%	0%	4%	3%	0%	5%	0%	0%	21%	0	0	0
Place innovation on fee for service lists/formularies	38%	6%	0%	0%	0%	6%	19%	0%	0%	7%	0%	2	4	6
Stage implementation scale up	38%	3%	4%	0%	0%	3%	4%	14%	0%	7%	3%	2	0	2
Change record system	37%	0%	0%	5%	4%	0%	4%	9%	9%	7%	0%	0	0	0
Change accreditation or membership reqs	37%	0%	0%	0%	8%	6%	15%	5%	0%	4%	0%	0	0	0
Use train the trainer strategies	34%	3%	0%	0%	8%	0%	4%	0%	9%	0%	10%	0	0	0
Use data experts	32%	6%	4%	0%	4%	3%	4%	0%	4%	7%	0%	0	0	0
Change physical structure and equipment	32%	0%	0%	0%	0%	0%	0%	32%	0%	0%	0%	0	0	0
Create new clinical teams	32%	0%	0%	10%	0%	0%	0%	9%	13%	0%	0%	0	0	0
Provide clinical supervision	29%	3%	0%	5%	0%	0%	0%	0%	4%	0%	17%	0	0	0
Develop an implementation glossary	27%	0%	4%	0%	4%	0%	4%	9%	0%	0%	7%	0	0	0
Develop disincentives	25%	0%	0%	0%	0%	6%	7%	5%	0%	7%	0%	0	0	0
Revise professional roles	25%	0%	0%	0%	0%	0%	4%	18%	0%	0%	3%	8	5	13
Develop and organize quality monitoring systems	22%	3%	0%	0%	0%	0%	15%	5%	0%	0%	0%	0	0	0
Use other payment schemes	22%	0%	0%	0%	0%	0%	15%	0%	0%	7%	0%	0	0	0
Change liability laws	22%	0%	0%	0%	0%	3%	19%	0%	0%	0%	0%	0	0	0
Change service sites	20%	3%	0%	0%	0%	0%	4%	14%	0%	0%	0%	0	0	0
Remind clinicians	11%	0%	0%	0%	0%	0%	0%	0%	4%	4%	3%	0	0	0
Make billing easier	11%	0%	0%	0%	0%	0%	7%	0%	0%	4%	0%	0	0	0
Alter patient/consumer fees	11%	0%	0%	0%	0%	3%	7%	0%	0%	0%	0%	0	0	0

**Alter patient/consumer fees**	11%	0%	0%	0%	0%	3%	7%	0%	0%	0%	0%	0	0	0
**Use data warehousing techniques**	8%	3%	0%	5%	0%	0%	0%	0%	0%	0%	0%	3	0	3
**Use capitated payments**	4%	0%	0%	0%	0%	0%	0%	0%	0%	4%	0%	0	0	0
